# The Canadian Medical Student Ultrasound Curriculum: A Statement From the Canadian Ultrasound Consensus for Undergraduate Medical Education Group

**DOI:** 10.1002/jum.15218

**Published:** 2020-01-13

**Authors:** Irene W. Y. Ma, Peter Steinmetz, Kirstin Weerdenburg, Michael Y. Woo, Paul Olszynski, Claire L. Heslop, Stephen Miller, Gillian Sheppard, Vijay Daniels, Janeve Desy, Maxime Valois, Luke Devine, Heather Curtis, Michael J. Romano, Patrick Martel, Tomislav Jelic, Claude Topping, Drew Thompson, Barbara Power, Jason Profetto, Pete Tonseth

**Affiliations:** ^1^ Division of General Internal Medicine University of Calgary Calgary Alberta Canada; ^2^ Department of Family Medicine McGill University Montreal Quebec Canada; ^3^ Department of Emergency Medicine McGill University Montreal Quebec Canada; ^4^ Department of Pediatric Emergency Medicine IWK Health Center and Dalhousie University Halifax Nova Scotia Canada; ^5^ Department of Emergency Medicine University of Ottawa and Ottawa Hospital Research Institute Ottawa Ontario Canada; ^6^ Department of Emergency Medicine University of Saskatchewan Saskatoon Saskatchewan Canada; ^7^ Division of Emergency Medicine, Department of Medicine University of Toronto Toronto Ontario Canada; ^8^ Division of General Internal Medicine University of Toronto Toronto Ontario Canada; ^9^ Department of Emergency Medicine, Skilled Clinician Program, Undergraduate Medical Education Dalhousie University Halifax Nova Scotia Canada; ^10^ Department of Diagnostic Imaging Dalhousie University Halifax Nova Scotia Canada; ^11^ Department of Emergency Medicine Memorial University of Newfoundland St John's Newfoundland Canada; ^12^ Division of General Internal Medicine University of Alberta Edmonton Alberta Canada; ^13^ Department of Emergency Medicine Sherbrooke University Sherbrooke Quebec Canada; ^14^ Department of Emergency Medicine, Thunder Bay Regional Health Sciences Center North Ontario School of Medicine Thunder Bay Ontario Canada; ^15^ Department of Emergency Medicine University of Manitoba Winnipeg Manitoba Canada; ^16^ Departments of Family Medicine and Emergency Medicine Laval University, Quebec Quebec Canada; ^17^ Department of Emergency Medicine Western University London Ontario Canada; ^18^ Department of Medicine, Education University of Ottawa Ottawa Ontario Canada; ^19^ Department of Family Medicine McMaster University Hamilton Ontario Canada; ^20^ Department of Radiology University of British Columbia Vancouver British Columbia Canada

**Keywords:** curriculum, education, point of care, ultrasound, undergraduate medical education

## Abstract

**Objectives:**

This study sought to establish by expert review a consensus‐based, focused ultrasound curriculum, consisting of a foundational set of focused ultrasound skills that all Canadian medical students would be expected to attain at the end of the medical school program.

**Methods:**

An expert panel of 21 point‐of‐care ultrasound and educational leaders representing 15 of 17 (88%) Canadian medical schools was formed and participated in a modified Delphi consensus method. Experts anonymously rated 195 curricular elements on their appropriateness to include in a medical school curriculum using a 5‐point Likert scale. The group defined consensus as 70% or more experts agreeing to include or exclude an element. We determined a priori that no more than 3 rounds of voting would be performed.

**Results:**

Of the 195 curricular elements considered in the first round of voting, the group reached consensus to include 78 and exclude 24. In the second round, consensus was reached to include 4 and exclude 63 elements. In our final round, with 1 additional item added to the survey, the group reached consensus to include an additional 3 and exclude 8 elements. A total of 85 curricular elements reached consensus to be included, with 95 to be excluded. Sixteen elements did not reach consensus to be included or excluded.

**Conclusions:**

By expert opinion‐based consensus, the Canadian Ultrasound Consensus for Undergraduate Medical Education Group recommends that 85 curricular elements be considered for inclusion for teaching in the Canadian medical school focused ultrasound curricula.

**Abbreviations**
CanUCMe: Canadian Ultrasound Consensus for Undergraduate Medical Education
UME: undergraduate medical education

Over the last decade, focused ultrasound has been increasingly integrated into the medical school curricula.^1^, ^2^, ^3^ Multiple schools have reported their experiences with teaching focused ultrasound in the undergraduate medical education (UME) setting. Focused ultrasound instruction in UME ranges from using ultrasound to facilitate the teaching of anatomy,^4^, ^5^, ^6^, ^7^, ^8^, ^9^ physical examination,^10^, ^11^, ^12^, ^13^, ^14^, ^15^ and procedural skills^16^, ^17^, ^18^ to schools that have comprehensively integrated focused ultrasound into their curricula.^19^, ^20^, ^21^, ^22^, ^23^, ^24^, ^25^ Given the broad scope of existing focused ultrasound applications, many educators struggle with deciding which focused ultrasound skills to include in the UME setting. Recommended curricula and educational strategies for medical schools have been previously published in Europe and the United States.^3^, ^26^, ^27^ Although it is appealing to adopt an existing recommended curriculum, the reality of medical education is that curriculum implementation is highly dependent on contextual limitations such as practice settings and the availability of human, infrastructure, and financial resources as well as expertise.^28^ Integrating focused ultrasound teaching into the UME curriculum requires an adequate infrastructure, time available in an already busy curriculum, and faculty resources.^29^, ^30^ To incorporate additional curricular items into an already full medical curriculum remains an additional challenge,^1^, ^30^ especially if the educational benefits have not always been consistently demonstrated.^31^, ^32^ Given these challenges, it is not surprising that despite positive learner experiences^33^, ^34^, ^35^ and consensus among many educators regarding the value and importance of teaching focused ultrasound,^1^, ^30^, ^36^ the adoption of focused ultrasound teaching into the UME setting continues to be variable.^37^ In a 2012 survey of US medical schools, only 62% of survey participants reported focused ultrasound integration into their UME curricula.^1^ In Canada, only 50% of the Canadian medical schools had implemented focused ultrasound education in a 2014 survey.^30^

Given feasibility concerns that exist in many medical school settings, focused ultrasound curriculum guidelines must be mindful of limitations and strengths specific to each medical school,^28^ and curriculum creation may be better served by focusing on teaching an achievable number of items of central importance,^28^ rather than a comprehensive list of applications. This study sought to establish a consensus‐based focused ultrasound curriculum for Canadian medical students.

## Materials and Methods

### *Study Group and Curricular Element Selection*

Ethical approval for this study was not sought for this consensus statement based on an A pRoject Ethics Community Consensus Initiative Ethics Screening Tool^38^ score indicating minimal risk. Each expert in our expert group verbally consented to participate in this consensus statement work.

The Canadian Ultrasound Consensus for Undergraduate Medical Education (CanUCMe) Group was formed in March 2018, comprising an expert panel of 21 focused ultrasound and educational leaders representing 15 of 17 (88%) Canadian medical schools. Individuals participating in the panel were identified on the basis of their focused ultrasound educational leadership roles within their medical schools. For medical schools whose focused ultrasound educational leaders were unknown to the group, we contacted the deans and associate deans of those medical schools to provide us with the contacts of their designated focused ultrasound educational leaders. Panel members participated in an introductory teleconference meeting on March 19, 2018, at which overarching principles used to guide curricular element selection were introduced and agreed on.^39^ Specifically, the group agreed that chosen curricular elements should be as follows^39^:

1. Selected on the basis of educational needs, clinical needs, or both;
2. Feasibly taught and learned to reflect the variability of resources available to teach focused ultrasound at each medical school; and
3. Based on clinical evidence, educational evidence, or both.

At the outset, our group sought to determine the minimum number of curricular elements that should be taught to ensure a foundational understanding of focused ultrasound, rather than a comprehensive list of topics that could be taught at a UME level.

### *Consensus Process*

Participants were asked to complete a baseline questionnaire capturing their ultrasound and medical education expertise. They then participated in a modified Delphi method by participating in anonymous iterative voting via an online survey platform (www.SurveyMonkey.com).^40^ We determined a priori that no more than 3 rounds of voting would be performed.

An initial survey consisting of 195 curricular elements was drafted on the basis of a review of relevant literature.^3^, ^26^, ^31^, ^33^, ^34^, ^35^, ^41^, ^42^, ^43^, ^44^, ^45^, ^46^, ^47^, ^48^ Articles deemed relevant to their curriculum development efforts were contributed by each member of the CanUCMe team and shared on an online platform (www.Dropbox.com) between March and June 2018. A draft survey was piloted with 5 focused ultrasound experts who were not part of the expert panel for feedback on items, wording, clarity, and flow. Before survey administration, the survey was also circulated to our expert panel for additional input.

In the first round of the survey conducted from August to September 2018, experts were asked to rate each curricular element on its appropriateness to include in a medical school curriculum using a 5‐point Likert scale, where 1 indicated very inappropriate to include; 3, neither appropriate nor inappropriate; and 5, very appropriate to include. Consensus to include an item was defined by 70% or more experts rating an item as 4 or 5. Consensus to exclude an item was defined by 70% or more experts rating an item as either 1 or 2. This 70% cutoff was consistent with current recommendations on consensus group methods.^40^ Items that did not reach consensus were readdressed in subsequent rounds. In the second and third rounds, participants were asked to consider each item in a binary fashion (yes, appropriate to include; versus no, not appropriate to include), and feedback on results from the prior round was provided to the participants in a percentage‐of‐agreement format. As in round 1, consensus was defined as 70% or more experts voting to include (or exclude) an item. Round 2 was conducted in December 2018, and round 3 was conducted 3 months later. The same experts were invited to participate in all rounds.

## Results

Baseline characteristics of our group of 21 experts are outlined in Table 1. All experts participated in all 3 survey rounds.

**Table 1:** Demographic Characteristics of the 21 Members of the CanUCMe Expert Panel Group

Characteristic	n (%)
Academic institution^a^	
University of British Columbia	1 (5)
University of Calgary	2 (10)
University of Alberta	1 (5)
University of Saskatchewan	1 (5)
University of Manitoba	1 (5)
Northern Ontario School of Medicine	1 (5)
Western University	1 (5)
McMaster University	1 (5)
University of Toronto	3 (14)
Queen's University	0
University of Ottawa	2 (10)
McGill University	2 (10)
University of Montreal	0
Sherbrooke University	1 (5)
Laval University	1 (5)
Dalhousie University	3 (14)
Memorial University of Newfoundland	1 (5)
Sex	
Male	14 (67)
Female	7 (33)
Specialty	
Emergency/pediatric emergency medicine	10 (48)
Family medicine	4 (19)
Internal medicine	5 (24)
Radiology	2 (10)
Experience in using ultrasound, y	
1–2	5 (24)
3–6	4 (19)
7–10	3 (14)
**≥**11	9 (43)
Experience in teaching ultrasound, y	
1–2	4 (19)
3–6	8 (38)
7–10	4 (19)
**≥**11	5 (24)
Experience in assessing ultrasound skills, y	
1–2 years	6 (29)
3–6 years	11 (52)
7–10 years	4 (19)
**≥**11	0
Specialized training in ultrasound and/or education	
Ultrasound fellowship training (**≥**1 y)	9 (43)
Graduate training in medical education (master's or PhD)	7 (33)

a Some individuals are cross‐appointed at more than 1 academic institution; therefore, the total exceeds 100%.

Of the 195 curricular elements considered in round 1, our group reached consensus to include 78 and exclude 24. Of the remaining 93 elements brought forward for consideration in round 2, our group reached consensus to include 4 and exclude 63. The remaining 26 elements were considered in round 3. On the basis of comments by the experts in round 2, given the difficulty in reaching consensus on specific procedural skills, experts recommended that an additional item (“general needle guidance technique using ultrasound”) be included in round 3. With these final 27 elements, for round 3, the group reached consensus to include 3 and exclude 8. There was consensus to not include 95 elements into the current Canadian UME curriculum (Table 2), and no consensus was reached for 16 elements (Table 3).

**Table 2:** Ninety‐Five Curricular Elements Reaching Consensus for Exclusion From Canadian UME and Round in Which Consensus Was Reached

Element for Exclusion	Round Reaching Consensus
Ultrasound concepts	
Advanced artifacts (eg, speed propagation artifact, slice thickness artifact)	2
Advanced knobology (eg, time‐gain compensation, harmonics)	2
Spectral Doppler imaging	1
Power Doppler imaging	2
Anatomy and physical examination	
Subclavian vein	3
Head and neck muscles	2
Esophagus	2
Lymph nodes	3
Intercostal vessels	2
Papillary muscles	3
Ascending thoracic aorta	3
Sternum/manubrium	2
Portal vein	2
Celiac artery	2
Superior mesenteric artery	2
Iliac artery	2
Splenic vein	2
Pancreas	2
Large bowel	2
Small bowel	2
Stomach	2
Ovaries	2
Prostate	2
Shoulder	2
Elbow	2
Wrist	2
Hands	1
Hip	2
Knee	2
Ankle	2
Feet	2
Median nerve	2
Ulnar nerve	2
Radial nerve	2
Femoral nerve	2
Sciatic nerve	2
Popliteal nerve	2
Tibial/peroneal nerve	2
Inguinal lymph nodes	2
Popliteal vessels	2
Dorsalis pedis	2
Achilles tendon	2
Quadriceps tendon	2
Physiology	
Baroreflex	2
Clinical applications	
Assessment of breast lesions	1
Apical 5‐chamber view	2
Suprasternal view	1
Right ventricular strain/dilatation	3
Ascending/thoracic aortic dissection	2
Left atrial enlargement	2
E‐point septal separation	3
Common bile duct measurements	1
Hepatomegaly/cirrhosis	2
Splenomegaly	2
Bowel obstruction	2
Pneumoperitoneum	1
Measuring fetal heart rate	3
Assessment of fetal lie	2
Measuring crown‐rump length	2
Assessment of amniotic fluid index	1
Use of transvaginal ultrasound	1
Testicular (eg, mass, hydrocele, torsion)	1
Pediatric: intussusception	1
Pediatric: pyloric stenosis	1
Pediatric: appendicitis	1
Pediatric: lymphadenitis	1
Hernia assessment (eg, inguinal, umbilical)	2
Deep venous thrombosis: lower extremity proximal	3
Deep venous thrombosis: lower extremity distal	2
Deep venous thrombosis: upper extremity	2
Soft tissue infections	2
Identifying shoulder effusions	2
Identifying hip effusions	2
Identifying elbow effusions	2
Identifying ankle effusions	2
Thyroid nodules	1
Intracranial Doppler	1
Retinal	2
Procedures	
Peripheral nerve block	2
Lumbar puncture	2
Intubation	2
Thyroid biopsies	1
Breast lesion biopsies	1
Solid‐organ biopsies	1
Lymph node biopsies	1
Joint arthrocentesis or steroid injections: shoulder	1
Joint arthrocentesis or steroid injections: knee	2
Joint arthrocentesis or steroid injections: hip	1
Joint arthrocentesis or steroid injections: other joints	1
Pericardiocentesis	2
Amniocentesis	1
Intrauterine device insertion	1
Format(s) of training	
Time spent with radiologists in the ultrasound department	2
Time spent with cardiologists	2
Time spent with obstetrics/gynecology	2

**Table 3:** Sixteen Curricular Elements That Did Not Reach Consensus for Either Inclusion or Exclusion From Canadian UME

Ultrasound concepts
Advanced control (eg, patient labeling)
Color Doppler imaging
Clinical applications
Acute cholecystitis findings
Ectopic pregnancy/confirming intrauterine pregnancy
Identifying yolk sac/gestational sac/fetal pole
Integrated scan protocols (eg, echo‐guided life support, cardiopulmonary limited ultrasound examination, bedside lung ultrasound in emergency, fluid administration limited by lung sonography, rapid ultrasound for shock and hypotension, etc)
Soft tissue infection (cellulitis, abscesses)
Identifying knee effusions
Procedures
Paracentesis
Thoracentesis
Central lines
Arterial line/arterial blood gas sampling
Abscess incision and drainage
Format(s) of training
Time spent with sonographers
Allow learners to scan themselves, unsupervised
Allow learners to scan each other, unsupervised

The final recommended curricular elements included 85 items (Table 4). All experts approved the final recommended curriculum.

**Table 4:** Final 85 Consensus‐Based Recommended Curricular Elements for Canadian UME and Round in Which Consensus Was Reached

Element for Inclusion	Round Reaching Consensus
Ultrasound concepts	
Ultrasound physics (eg, frequency, wavelengths)	1
Sound interactions with tissue (eg, reflection, scatter, refraction)	1
Common artifacts (eg, reverberations, attenuation, shadowing, post–acoustic enhancement)	1
Basic knobology (eg, depth, gain)	1
Primary control (eg, freeze, save images/cine loops)	1
B‐mode imaging	1
M‐mode imaging	1
Transducer characteristics	1
Transducer orientation	1
Scan plane terminology (eg, coronal, sagittal, axial)	1
Transducer movements (eg, sliding, heel‐toeing/rocking)	1
Basic ultrasound terminology (eg, anechoic, hyperechoic, complex, heterogeneous)	1
ALARA (as low as reasonably achievable) principle	1
Potential bioeffects (eg, thermal, mechanical)	3
Patient interactions	
Obtain consent	1
Appropriate hand hygiene and infection control practices	1
Appropriate patient interaction	1
Appropriate patient draping	1
Appropriate management of incidental findings	1
Appropriate communication of findings including uncertainties	1
Recognize scope, limitations, and when to ask for help	1
Anatomy and physical examination	
Thyroid	1
Internal jugular vein	1
Carotid artery	1
Trachea/thyroid cartilage	1
Ribs	1
Pleura	1
Diaphragm	1
Right ventricle	1
Left ventricle	1
Left atrium	1
Right atrium	1
Interventricular/interatrial septum	1
Cardiac valves (eg, aortic, mitral, tricuspid)	1
Cardiac apex	1
Pericardium	1
Liver	1
Spleen	1
Kidneys	1
Aorta	1
Inferior vena cava	1
Spine	1
Gallbladder	2
Urinary bladder	1
Uterus	1
Proximal inguinal regional vessels (eg, femoral artery/vein/great saphenous)	1
Physiology	
Cardiac cycle	1
Heart sound generation	1
Systole/diastole	1
Clinical applications	
Recognition of appropriate indications for point‐of‐care ultrasound use	1
Sources of false‐positive and false‐negative results	1
Implications of presence of false‐positive and false‐negative results on clinical decision making	1
Appropriate application of evidence regarding indications/image acquisition/image interpretation issues into specific patient contexts	1
Recognition of cystic vs solid/noncystic structures	1
Normal lung (A lines)	1
B lines/interstitial syndrome	1
Pleural effusion	1
Consolidation	1
Pneumothorax	1
Parasternal long‐axis view	1
Parasternal short‐axis view	1
Apical 4‐chamber view	1
Subcostal 4‐chamber view	1
Gross left ventricular function	1
Pericardial effusion	1
Free fluid: right upper quadrant	1
Free fluid: left upper quadrant	1
Free fluid: pelvic views	1
Hydronephrosis	1
Abdominal aortic aneurysm	1
Inferior vena cava	1
Jugular venous height	1
Procedures	
Ultrasound‐guided peripheral intravenous insertion	1
General needle guidance technique using ultrasound	3
Recommended format(s) of training	
Use of didactic lectures	2
Use of small‐group scanning on standardized patients	1
Use of small‐group scanning on patients	1
Use of online videos/podcasts	1
Use of simulation	1
Use of interprofessional training	2
Time spent with point‐of‐care ultrasound providers	1
Use of student interest groups	1
Use of peer teachers	1
Allow learners to scan themselves, supervised	3
Allow learners to scan each other, supervised	2

## Discussion

Our expert panel reached consensus that 85 focused ultrasound curricular elements can be considered for implementation in the Canadian UME curriculum. Of the initial 195 items considered, there was also general consensus to exclude 95 (49%) items. Only 16 items (8%) did not reach consensus after 3 rounds. For items on basic ultrasound concepts and patient interactions, our experts readily reached consensus. However, our experts were not able to reach consensus on many of the ultrasound‐guided procedures. Currently, required procedural competencies vary among residencies; there is no single list of mandatory bedside procedures that all Canadian medical students are expected to master before graduation.^49^ Therefore, our inability to reach consensus on which ultrasound‐guided procedures to include may have been more a function of having no prior consensus on procedural expectations than a lack of consensus regarding the ultrasound component itself, especially with our panel of diverse specialists.

Our recommended curriculum differs from existing national curricula in a number of ways.^3^, ^26^, ^27^ First, we used explicit consensus‐based methods to achieve our list of agreed‐on curricular elements.^40^ Second, we solicited broad‐based representation from focused ultrasound education leaders across the country and from a variety of specialties. Our panel was composed of leaders from more than 80% of Canadian medical schools; this representation and involvement of key stakeholders ensure that our recommendations are relevant across medical schools and will facilitate future implementation processes. Third, at the outset, the group was tasked with the development of a minimum number of curricular elements, keeping in mind the clinical and educational needs and evidence, as well as issues regarding educational feasibility in the Canadian medical school environment. Similar to existing curricula, we expect that variations will occur in the curriculum implementation processes across the country because of local resource and contextual differences among schools.^26^ Our curriculum is intended as a guide: recommended elements are suggested, but not considered mandatory, and excluded elements are not prohibited.

Our study had several limitations. First, our panel was composed entirely of Canadian experts, and our target audience was Canadian medical students, which limit the generalizability of our suggested curricular topics to countries where the educational context, resources, and expertise are similar. Second, whereas our experts took feasibility into consideration in designing the curriculum, whether this curriculum is indeed feasible at all Canadian medical schools remains to be seen. Third, our panel did not involve learners, patient representatives, and other stakeholders; student, patient, and other stakeholder engagement will be an important part of successful curriculum implementation.^50^ Fourth, our curriculum does not explicitly address competency‐based requirements or assessment processes for each element. For example, for the clinical application on the assessment of gross left ventricular function, we have not specified exact methods for estimating function, nor have we specified how competency in this skill is to be defined. Addressing focused ultrasound skill competency will become increasingly important as learner levels progress. At one end of the spectrum, learners advance from the medical student stage at which ultrasound is used as an educational tool, and clinical practice is substantially supervised. In contrast, postgraduate medical education training and independent practice involve a skill set that integrates focused ultrasound findings into clinical decision making. In addition, we have not provided details on our included curricular elements. For example, for the evaluation of pleural effusions, we have not specified whether individual schools should teach methods for estimating the size of pleural effusions. Future work could further clarify curricular details. Fifth, it is important to emphasize that our recommended curriculum is based on expert opinion‐based consensus and not an evidence‐based literature review. Although our panel was composed of education experts familiar with focused ultrasound education, and a number of evidence‐based systematic and scoping reviews were used as a basis of our survey, we did not conduct a systematic review ourselves. Last, despite a diverse list of specialty involvement, our panel was composed of clinicians, and 48% of our experts were emergency medicine specialists. Because of our inclusion criteria, we did not have representation from anatomists, physiologists, pathologists, and specialties such as surgery, obstetrics and gynecology, neurology, pediatrics, and anesthesiology. Our experts were those charged with leading focused ultrasound teaching for each of the medical schools, and currently in Canada, these roles are primarily filled by clinicians. Additional input from basic scientists and other specialties not represented in our panel would be valuable and should be included in curriculum design and implementation processes.

In conclusion, the CanUCMe Group recommends that 85 curricular elements be considered for inclusion into the Canadian medical school focused ultrasound curriculum. We believe that these proposed elements can assist UME trainees in attaining a uniform and strong foundational understanding of focused ultrasound concepts and techniques.
